# Treg Depletion Inhibits Efficacy of Cancer Immunotherapy: Implications for Clinical Trials

**DOI:** 10.1371/journal.pone.0001983

**Published:** 2008-04-23

**Authors:** James F. Curtin, Marianela Candolfi, Tamer M. Fakhouri, Chunyan Liu, Anderson Alden, Matthew Edwards, Pedro R. Lowenstein, Maria G. Castro

**Affiliations:** 1 Department of Biomedical Sciences, Gene Therapeutics Research Institute, Cedars Sinai Medical Center, Los Angeles, California, United States of America; 2 Department of Medicine, The Brain Research Institute, and Jonsson Comprehensive Cancer Center, David Geffen School of Medicine, University of California Los Angeles, Los Angeles, California, United States of America; 3 Department of Molecular and Medical Pharmacology, The Brain Research Institute, and Jonsson Comprehensive Cancer Center, David Geffen School of Medicine, University of California Los Angeles, Los Angeles, California, United States of America; City of Hope Medical Center and Beckman Research Institute, United States of America

## Abstract

**Background:**

Regulatory T lymphocytes (Treg) infiltrate human glioblastoma (GBM); are involved in tumor progression and correlate with tumor grade. Transient elimination of Tregs using CD25 depleting antibodies (PC61) has been found to mediate GBM regression in preclinical models of brain tumors. Clinical trials that combine Treg depletion with tumor vaccination are underway to determine whether transient Treg depletion can enhance anti-tumor immune responses and improve long term survival in cancer patients.

**Findings:**

Using a syngeneic intracrabial glioblastoma (GBM) mouse model we show that systemic depletion of Tregs 15 days after tumor implantation using PC61 resulted in a decrease in Tregs present in tumors, draining lymph nodes and spleen and improved long-term survival (50% of mice survived >150 days). No improvement in survival was observed when Tregs were depleted 24 days after tumor implantation, suggesting that tumor burden is an important factor for determining efficacy of Treg depletion in clinical trials. In a T cell dependent model of brain tumor regression elicited by intratumoral delivery of adenoviral vectors (Ad) expressing Fms-like Tyrosine Kinase 3 ligand (Flt3L) and Herpes Simplex Type 1-Thymidine Kinase (TK) with ganciclovir (GCV), we demonstrate that administration of PC61 24 days after tumor implantation (7 days after treatment) inhibited T cell dependent tumor regression and long term survival. Further, depletion with PC61 completely inhibited clonal expansion of tumor antigen-specific T lymphocytes in response to the treatment.

**Conclusions:**

Our data demonstrate for the first time, that although Treg depletion inhibits the progression/eliminates GBM tumors, its efficacy is dependent on tumor burden. We conclude that this approach will be useful in a setting of minimal residual disease. Further, we also demonstrate that Treg depletion, using PC61 in combination with immunotherapy, inhibits clonal expansion of tumor antigen-specific T cells, suggesting that new, more specific targets to block Tregs will be necessary when used in combination with therapies that activate anti-tumor immunity.

## Introduction

Glioblastoma multiforme (GBM) is a deadly primary brain tumor which is highly invasive with tumor cells infiltrating the surrounding healthy brain tissue [1]. The median survival of patients diagnosed with GBM is one year (4–6 months after recurrence), with less than 5% of the patients remaining alive 5 years after diagnosis [2]. Improvements in surgery, chemotherapy and radiotherapy have not been translated into significantly improved prognosis for patients with GBM; long term survival (5 years after diagnosis) has not improved since 1950 [3]. Tumor recurrence almost always occurs even if surgery successfully removes the majority of the primary tumor mass. Novel therapies to prevent or treat tumor recurrence are urgently needed to treat patients diagnosed with GBM.

Immunotherapy has been proposed as a powerful approach to prevent tumor recurrence by eliminating tumor cells while sparing normal surrounding healthy cells [4], [5]. Several clinical trials are now underway to test whether immunotherapy is safe and effective to treat GBM [6], [7]. GBMs over express tumor antigens such as MAGE, Her2/neu, Tyrosinase, Trp-1, Trp-2, gp100, IL13Rα2, Survivin (reviewed in [8]) and EphA2 [9]. The immune system ordinarily sculpts tumors resulting the loss of tumor antigen expression [10], [11], however, the location of GBM in the brain, a site of immune privilege [12], [13], or the presence of a highly immunosuppressive environment in brain tumors [14], [15] may be reasons why GBM commonly over express tumor antigens in patients. Autologous dendritic cells (DC) loaded with GBM tumor peptides [16] or autologous tumor lysate [17] have been used to vaccinate patients in two recent Phase I clinical trials. No significant increase in survival was observed using autologous tumor lysates [17]. However, the median time to progression and median survival of patients treated with peptide based vaccines was increased compared with patients that were treated during the same time period with conventional therapies [16]. Interestingly, a subpopulation of responders to the treatment were identified by the expression of low concentrations of TGFβ in the brain. Intratumoral expression of TGFβ can suppress adaptive immune responses against antigen [4], [5] and was predictive of clinical outcome after vaccination [16]. In addition, circulating tumor antigen specific CD8^+^ T lymphocytes have been identified in GBM patients [18], but the immunosuppressive environment in the tumor prevents the elimination of GBM from these patients.

T cell responses against tumor antigen measured by tetramers and ELISPOT do not always correlate with tumor regression in clinical trials testing immunotherapies for human GBM [19]. This suggests that suppression of effective immune responses against tumor antigens can interfere with immune dependent tumor regression. Recently, researchers have investigated whether depletion of a subset of T lymphocytes called regulatory T lymphocytes (Tregs) can potentiate immunotherapies against cancer. Tregs are a subpopulation of CD4^+^ T lymphocytes that constitutively express the transcription factor Foxp3, the high affinity IL2 receptor CD25 and the B7 ligand CTLA4 [20]. Tregs are required for the maintenance of tolerance throughout the lifetime of the organism [21] and mutations in Foxp3 are known to cause acute autoimmune disorders in humans [22]. Foxp3^+^ Tregs accumulate within human gliomas during tumor progression[23] and have been found to correlate with tumor grade [24]. Survival was improved when Tregs were depleted from GL261 tumor bearing mice using CD25 antibodies administered 7 days after tumor implantation and three times per week for 3 weeks thereafter [25]. Also, administration of CD25 depleting antibodies were also shown to improve survival when administered in combination with DC transfected with tumor mRNA in a GBM model [26].

We have recently developed a combined gene therapeutic approach aimed at engineering the tumor microenvironment to induce the migration into the tumor mass of APCs and elicit tumor cell death [27]. Our approach consists of expressing Fms-like tyrosine kinase 3 ligand (Flt3L) which induces dendritic cell (DC) infiltration into the brain parenchyma [28] in combination with the conditional cytotoxic gene Thymidine Kinase (TK) [27], [29], which in the presence of GCV induces tumor cell death. This combined approach induces T cell dependent tumor regression [27]. We wished to assess the effects elicited by depletion of Tregs; on tumor progression in untreated GBM bearing mice and on T cell-dependent brain tumor regression elicited by Ad-Flt3L and Ad-TK treatment. Our data demonstrated that depletion of Tregs using the CD25 specific hybridoma PC61 induced tumor regression and long term survival when administered 15 days after tumor implantation, independent of tumor antigen specific T lymphocytes. In combination with intratumoral delivery of Ad-Flt3L and Ad-TK, however, we found that PC61 administration 24 days after tumor implantation (7 days after treatment) completely suppressed adaptive immune responses against GL26 tumor antigens. In addition to Tregs, CD25 is also expressed on the cell surface of precursor B and T lymphocytes and a mature subpopulation of DC [30] and is also upregulated on the cell surface of activated T lymphocytes and B lymphocytes [31]. In our model, administration of CD25-antibodies also eliminated activated, CD25^+^ T lymphocytes and prevented clonal expansion of tumor antigen specific T cells. Our data suggest that the use of PC61 aiming at Treg depletion could reduce the efficacy of immunotherapeutic regimes by suppressing activation and clonal expansion of tumor antigen specific T lymphocytes.

## Results

### Syngeneic intracranial GBM tumors are densely infiltrated with Immune cells, including Tregs

**Figure 1:**
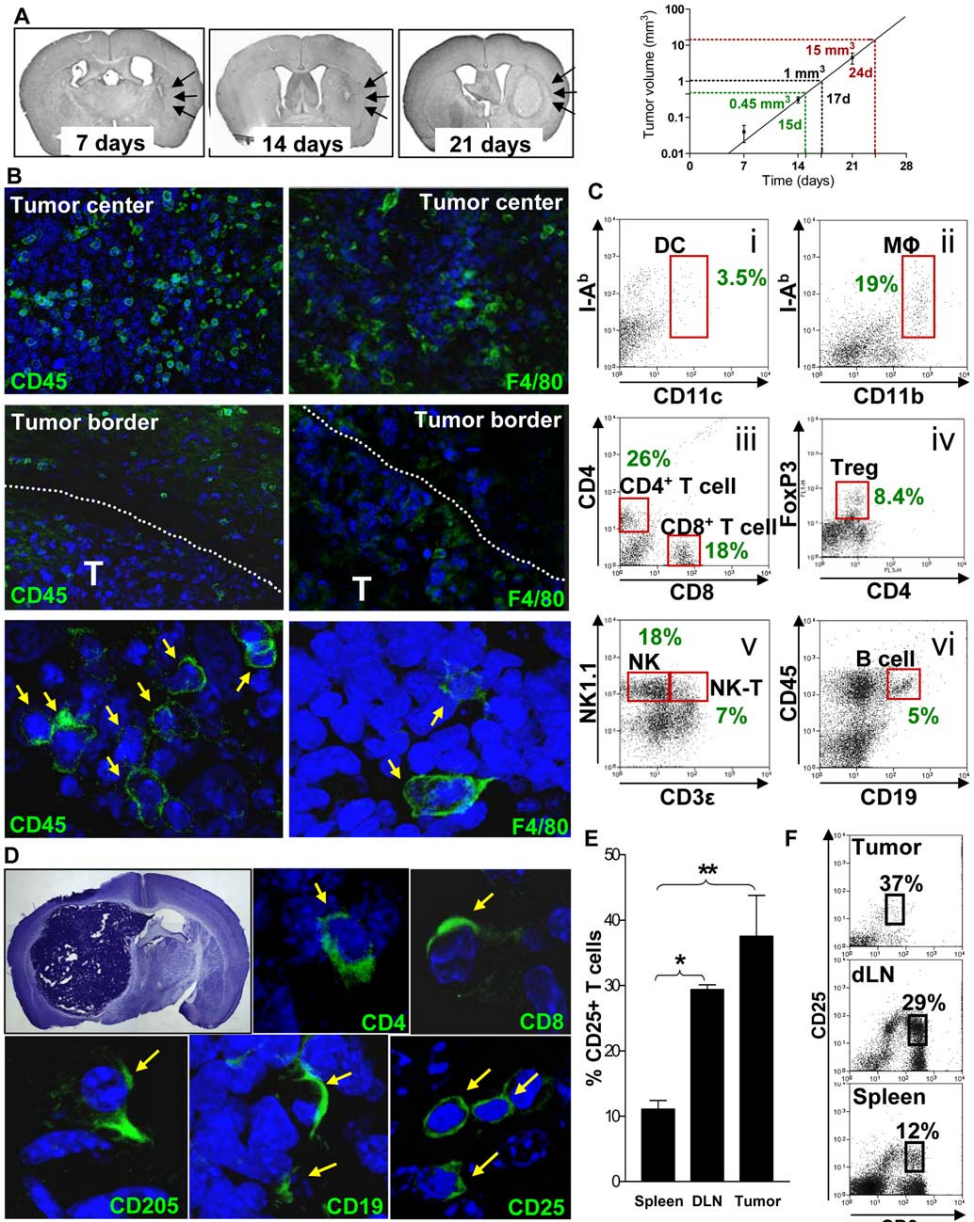
Intracranial GBM tumors are densely infiltrated with immune cells. (A) GL26 tumor volume 7, 14 and 21 days after implantion into the brain striatum of C57BL/6 mice was determined. Reactive astrocytic staining (GFAP+) was used to define the border of the tumor with non-malignant brain tissue. Representative sections of mouse brains bearing tumors implanted 7, 14 and 21 days previously and stained with GFAP are shown on the left. Tumor growth kinetics were essentially exponential over the time period analyzed with a doubling time of approximately 1.8 days. 5 mice were used to determine tumor volume at each time point. (B) Infiltration of immune cells in intracranial GL26 tumors. Brain sections from tumor bearing mice were stained with antibodies against CD45 (leukocytes) or F4/80 (macrophages/activated microglia). Representative Confocal images show immune cells (green) within the tumor mass and in the tumor borders. DAPI (blue) was used to stain nuclei. Yellow arrows indicate immunoreactive cells. T: tumor. (C) Tumor infiltrating immune cells were isolated from tumors 24 days after tumor implantation and analyzed using flow cytometry. (i) Dot plot of CD11c against I-Ab that was first gated for live CD45+ leukocytes. DC (CD11c+ CD45+ I-Ab+) are shown in the red box. (ii) Macrophages (MΦ) were assessed by gating live leukocytes with CD45, then plotting CD11b against I-Ab. The red box outlines the population of tumor infiltrating macrophages (CD11b+ CD45+ I-Ab+). (iii) T cells were stained with CD3ε-PE, CD4-PerCP and CD8a-FITC. The plot displays CD4 against CD8a when cells were first gated for CD3ε+ live leukocytes. CD4+ T cells and CD8a+ T cells are shown in red boxes. (iv) Tumor infiltrating Tregs were observed by staining with CD3ε-PE, CD4-PerCP and Foxp3-FITC. CD3ε+ live leukocytes were gated and dot plots display Foxp3 against CD4 staining. The population of Tregs (CD4+ Foxp3+ CD3ε+) are shown in the red box. (v) NK and NK-T cells were visualized by gating live CD45+ leukocytes, then displaying NK1.1 against CD3ε. The population of tumor infiltrating NK cells (CD3ε- CD45+ NK1.1+) and NK-T cells (CD3ε+ CD45+ NK1.1+) are shown in red boxes. (vi) Tumor infiltrating B cells are visualized by gating for live leukocytes, then plotting CD45 against CD19. The population of B cells (CD19+ CD45+) is shown in a red box. The percentages of each immune cell population infiltrating the tumor with respect to the total number of tumor CD45+ cells is indicated in representative dot plots. (D) Nissl staining was used to visualize tumors in the brain. Tumors are dense in Nissl substance, so stain darker than normal brain tissue. Representative Confocal images show tumor infiltrating CD4+ T cells, CD8+ T cells, CD205+ mDCs, CD19+ B cells, and CD25+ immune cells (seen in green) and DAPI (blue) was used to visualize the nuclei. Yellow arrows indicate immunoreactive cells. (E) Flow cytometric analysis of the percentage of T cells that express CD25 in spleens, dLN and tumors in mice bearing intracranial GL26 brain tumors 24 days after tumor implantation. (F) Representative dot plots of CD25 vs CD3ε in immune cells isolated from the spleen, LN and tumor. The percentages of CD25+ cells population with respect to the total number of CD3ε+ cells in the tumor, draining lymph nodes or spleen are indicated in representative dot plots.

Accumulation of Foxp3^+^ Tregs in human gliomas correlates with the grade of the tumor and patient survival [24]. Accumulation of Foxp3^+^ Tregs in syngeneic mouse gliomas has also been described and depletion of Tregs with CD25-specific immunoglobulins can induce tumor regression and improve survival in these preclinical models [25], [32], [33]. In order to investigate whether Treg depletion could improve therapeutic outcome in combination with immunotherapy/gene therapy, we first established whether Tregs infiltrated into the syngeneic mouse glioma arising from the intracranial implantation of GL26 cells into the brain striatum. Implantation of syngeneic GL26 cells reproducibly led to the linear growth of tumors (R^2^>0.99) with a mean tumor volume of approximately 0.5mm^3^ at day 15 and 30 times larger (15mm^3^) at day 24 (Fig. 1A). These tumors exhibit profuse infiltration of CD45^+^ and F4/80^+^ cells, which are evident throughout the tumor mass and also at the borders (Fig 1B). Immune cell infiltration into the tumors was also assessed by flow cytometry 24 days after tumor implantation (Fig. 1 C). We detected tumor infiltrating mDC (CD11c^+^ CD45^+^ I-A^b+^, Fig. 1Ci) and MΦ (CD11b^+^ CD45^+^ I-A^b+^, Fig. 1Cii) that represented 3.5% and 19% of the total number of CD45+ immune cells present in the tumor respectively. Lymphocytes were also identified and constituted ∼80% of the total number of CD45+ immune cells infiltrating the tumors (Fig. 1 C iii-vi). These included CD4^+^ T cells (CD3ε^+^ CD4^+^ CD8a^−^, 26%), CD8a^+^ T cells (CD3ε^+^ CD4^−^ CD8a^+^, 18%), Foxp3^+^ Tregs (CD3ε^+^ CD4^+^ Foxp3^+^, 8.4%), NK cells (CD3ε^−^ NK1.1^+^ CD45^+^, 18%), NK-T cells (CD3ε^+^ NK1.1^+^ CD45^+^, 7%) and B cells (CD3ε^−^ CD19^+^ CD45^+^, 5%). The presence of CD4^+^, CD8a^+^, CD19^+^ lymphocytes as well as CD205 mDC were also observed by immunofluorescence (Fig. 1 D). In addition, large numbers of lymphocytes that were immunoreactive for CD25 were observed within the tumor using confocal microscopy (Fig. 1 D) and flow cytometry (CD3ε^+^ CD25^+^ CD45^+^) (Fig. 1 E, F). Moreover, the percentage of CD3ε^+^ T cells that expressed cell surface CD25 was ∼40% of the total number of tumor infiltrating T cells and was greatly elevated in the draining (cervical) lymph node (dLN) (p<0.05) and tumor (p<0.01) when compared with the spleen of tumor bearing mice (Fig. 1 E).

### Depletion of Tregs using PC61 induces brain tumor regression but also reduces T cells in tumor and dLN

**Figure 2:**
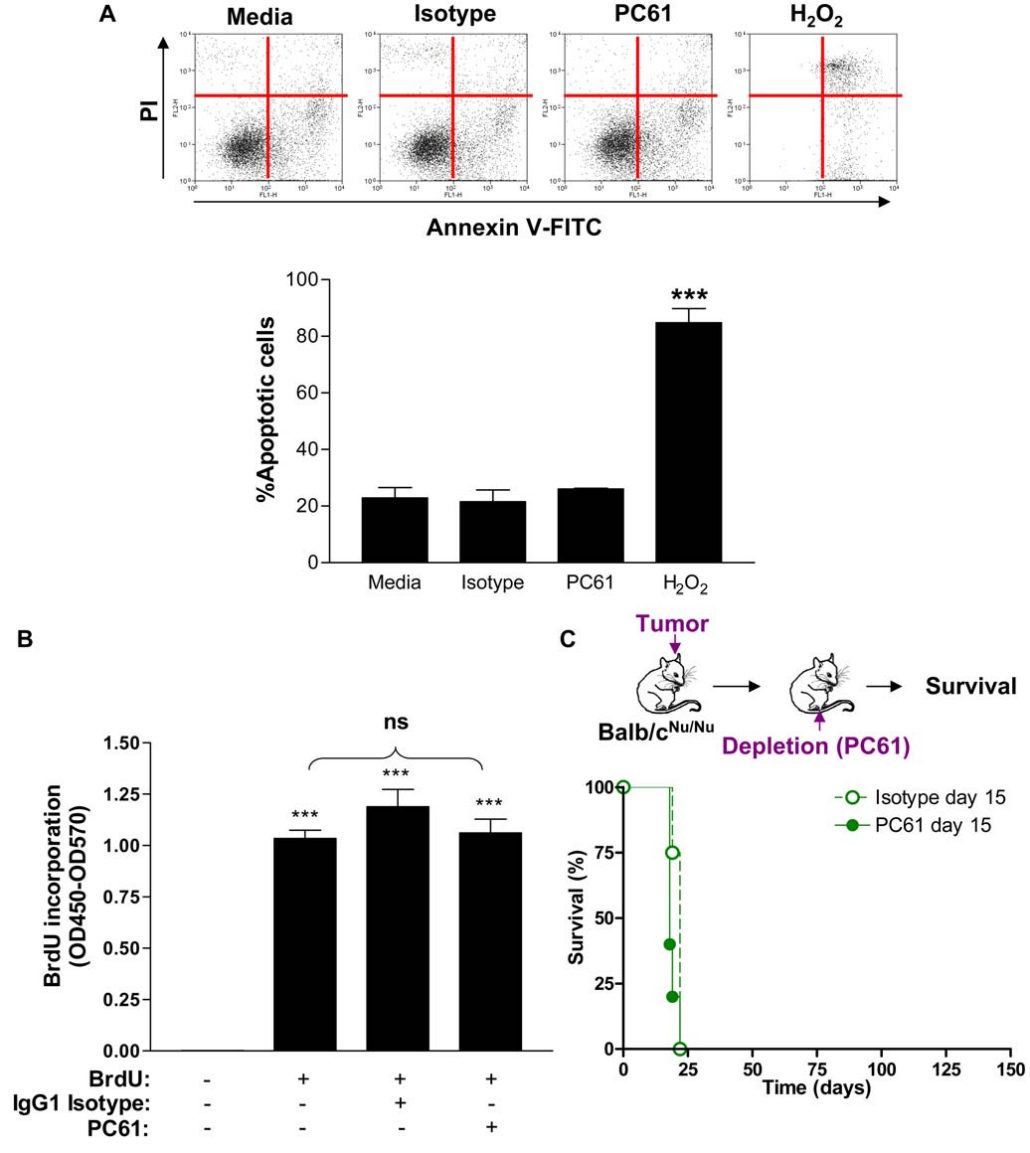
PC61 does not affect the viability or proliferation of GL26 cells *in vitro* or *in vivo*. (A) GL26 cells were incubated for 48 h with 100 µg/ml PC61 or 100 µg/ml rat IgG1 isotype control. Cells were then harvested and stained with Annexin V-FITC and Propidium Iodide to identify apoptotic cells. No significant increase in apoptosis was noted when cells were incubated with PC61 or isotype control immunoglobulins compares with untreated controls (p>0.05). A large increase in apoptosis was observed when cells were incubated for 4 h with H2O2 (p<0.001). Error bars represent the standard error from 3 replicates and the assay was repeated 3 times. One Way ANOVA with Tukey's post test was used to calculate significant differences (***p<0.001). (B) Proliferation of GL26 cells was measured by detecting BrdU incorporation into DNA. No significant difference in proliferation was observed (p>0.05) when cells were incubated with PC61 (rat IgG1αCD25) compared with isotype controls (rat IgG1), or untreated cells (***p<0.001 compared to negative (no cells) control, ns = non significant). (C) Athymic nu/nu mice (Balb/c background) were challenged with a single intracranial injection of GL26 cells and treated 15 d later with 1mg either PC61 or isotype control immunoglobulins injected i.p. Survival was monitored and mice were euthanized when moribund. No significant difference in survival was observed using a log rank test (p>0.05) (n = 5).

Considering that we detected extensive infiltration of T regs within the intracranial GL26 tumors, we aimed to deplete this immune cell population in order to determine their effect on tumor cell progression and on anti-tumor immune response induced by a T-cell dependent immunotherapeutic approach, i.e. Ad-Flt3L/TK. The rat IgG1 hybridoma PC61 was found to bind with mouse high affinity IL-2 receptor (CD25) [34] and *in vivo* administration of PC61 to mice causes the depletion of Tregs for more than a week [33], [35]. Thus, we used this antibody to induce T reg depletion *in vivo* in a syngeneic mouse intracranial glioma model. We first determined whether PC61 would affect tumor cell viability and growth *in vitro* and *in vivo*. Incubation of GL26 cells with PC61 did not affect GL26 cell viability (Fig. 2A) or proliferation *in vitro* (Fig. 2B). We then administered PC61 or isotype control IgG to tumor-bearing athymic nu/nu mice, which have been shown to be deficient in T regs [26], [36]. Tallying with other reports [26], we found that tumor growth in T reg deficient *nu/nu* immune deficient mice was not affected by PC61; both isotype control or PC61-injected mice succumbed due to tumor burden 18-22 days after tumor implantation.

**Figure 3:**
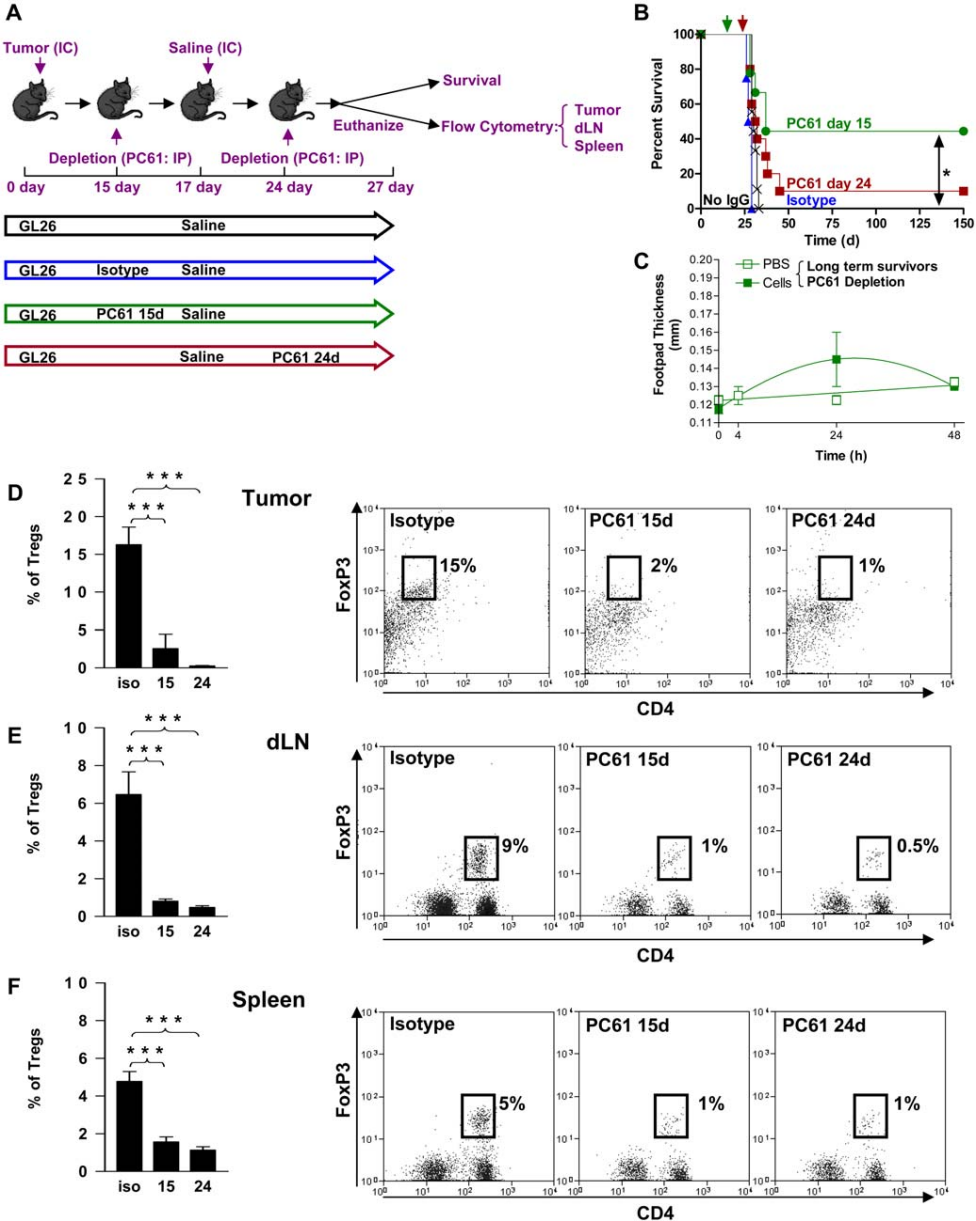
Depletion of Tregs 15 days post-tumor implantation induces brain tumor regression. (A) Diagram depicting the different treatments administered to 4 cohorts of tumor bearing mice. GL26 cells were implanted in the brain of all mice on day 0 and were treated by the intratumoral delivery of saline on day 17 with depletion of CD25^+^ cells using PC61 on day 15 (green) or on day 24 (red). Control groups of tumor bearing mice received rat IgG1 isotype control on day 15 (blue), or no systemic administration of immunoglobulins (Black) as indicated. Mice were used either for survival studies, or euthanized at day 27 post-tumor implantation for analysis of immune cell populations by flow cytometry. (B) Kaplan-Meier curve displaying survival of the 4 groups of mice described in (A). Ten mice were used in each group and a log-rank test was used to calculate significance between untreated group and PC61 treated groups. *p<0.05 compared with untreated mice and isotype control treated mice. (C) DTH test was performed on long-term survivor mice 100 d after tumor implantation. Mice had originally been treated with PC61 on day 15 (green lines). Irradiated GL26 cells in PBS in the right rear footpad (filled box) or PBS control in the left rear footpad (open box) of mice. The thickness of the footpad was determined 0 h, 4 h, 24 h and 48 h after injection of irradiated cells or PBS. Two Way ANOVA with Tukey's post test was used to calculate significant differences. (D–F) Tregs (CD3ε^+^ CD4^+^ Foxp3^+^) infiltrating tumors (D), draining lymph nodes (E) or spleens (F) were quantified in mice administered rat IgG1 isotype control (iso) or PC61 on day 15 (15) or day 24 (24) as indicated in the graphs (left). Dot plots (right) display representative data from 5 mice per group. The percentages of T regs with respect to the total number of CD45+ cells in the tumors, draining lymph nodes (dLN) or spleen are indicated in representative dot plots. One Way ANOVA with Tukey's post test were used to determine significant differences (***p<0.001).

**Figure 4:**
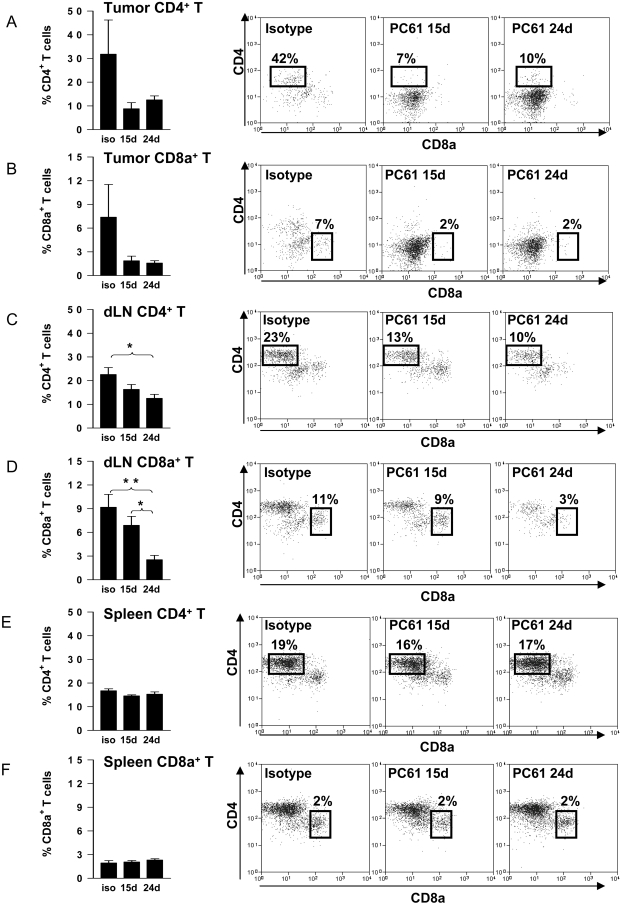
Infiltrating CD4+ T cells and CD8a+ T cells decrease in the tumor and dLN after treatment with PC61. C57BL/6 mice were challenged with tumor cells on day 0, and treated on day 17 by intratumoral delivery of saline. Tregs were depleted on either day 15 or day 24 by ip injection of PC61. Immune cells were isolated from the spleen, the cervical LN and the intracranial tumor at day 27 post-tumor implantation to determine the percentage of CD4+ T cells and CD8a+ T cells in each tissue. The percentage of tumor infiltrating immune cells that were CD4+ T cells (CD3ε+ CD4+ CD8a-) (A) and CD8a+ T cells (CD3ε+ CD4- CD8a+) (B); the percentage of immune cells in the draining lymph nodes that were CD4+ T cells (CD3ε+ CD4+ CD8a-) (C) and CD8a+ T cells (CD3ε+ CD4- CD8a+) (D); and the percentage of immune cells in the spleen that were CD4+ T cells (CD3ε+ CD4+ CD8a-) (E) and CD8a+ T cells (CD3ε+ CD4- CD8a+) (F) are shown in graphs (left). Representative dot plots for each treatment group are displayed on the right (gated for live leukocytes with FSC vs SSC and CD3ε+ T lymphocytes). The percentages of each immune cell population with respect to the total number of CD45+ cells in the tumors, draining lymph nodes (dLN) or spleen are indicated in representative dot plots. One Way ANOVA with Tukey's post test were used to calculate significant differences. (*p<0.05, **p<0.01).

**Figure 5:**
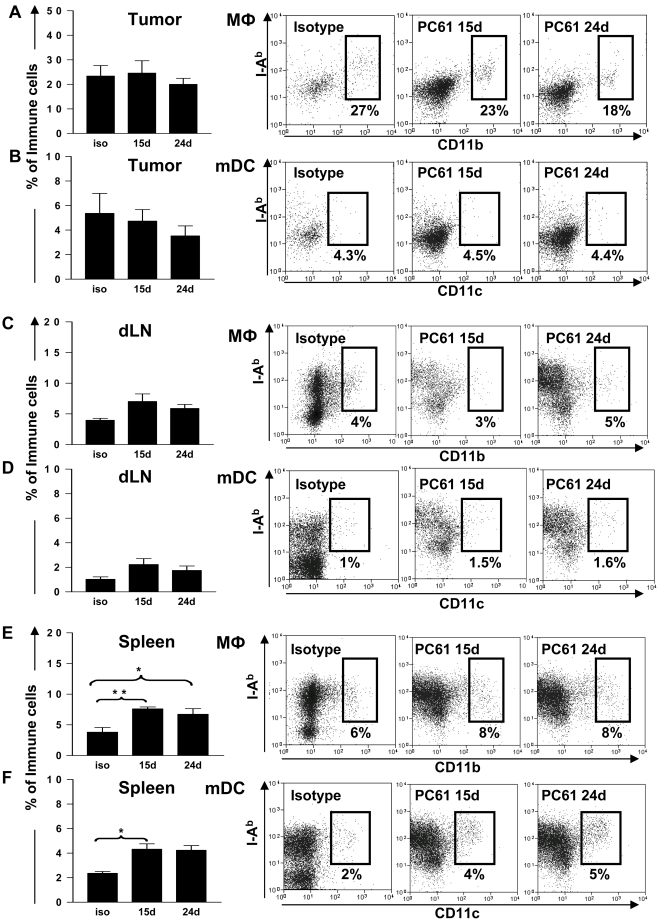
Antigen presenting cells do not change in tumor or dLN in untreated animals but increase in spleen after depletion of Tregs with PC61. GL26 cells were implanted in the brain striatum of C57BL/6 mice. Rat IgG1 isotype control immunoglobulins (-) were administered on day 15 and PC61 was administered on day 15 or day 24 as indicated. The percentage of tumor infiltrating macrophages (MΦ; CD11b+ CD45+ I-Ab+) (A,C,E) and myeloid Dendritic cells (mDC; CD11c+ CD45+ I-Ab+) (B,D,F) were quantified at day 27 post-tumor implantation using flow cytometry in the tumor (A,B) dLN (C,D) and spleen (E,F). The percentages of each immune cell population with respect to the total number of CD45+ cells in the tumors, draining lymph nodes (dLN) or spleen are indicated in representative dot plots. 5 and 10 mice were analyzed per group. One Way ANOVA with Tukey's post test was used to determine statistical significance.

After ruling out any direct effect of PC61 on tumor cell growth we studied the effect of T reg depletion in wild type C57/B6 mice bearing intracranial GL26 glioma. The experimental paradigm used is depicted in Fig. 3A. Depletion of Tregs 15 days after tumor cell implantation significantly increased the number of long term survivors with 40% of mice were still alive 150 days later (p<0.05, Fig. 3B). This was more than 5 times longer than the mean survival of control tumor bearing mice (Fig. 3B). No significant increase in long term survival was observed when Tregs were depleted in larger tumors (24 days) (p>0.05) and only 10% of mice survived long term from this group (Fig. 3B). Interestingly, we did not observe any DTH responses when we injected irradiated GL26 cells into the footpad of long term survivors from Treg depleted mice (Fig. 3C). In order to assess the effects of treatment with PC61 *in vivo*, we analyzed the presence of Foxp3^+^ CD4^+^ Tregs infiltrating the GBM mass as well as in the draining lymph nodes and the spleen after intraperitoneal injection of PC61 (days 15 and 24) or the rat IgG1 isotype control (day 15 days) into tumor bearing mice. PC61 injection on day 15 led to a 9 fold reduction (p<0.001) in the number of tumor infiltrating Tregs when measured using flow cytometry 12 days later (day 27), and injection of PC61 on day 24 led to the almost complete depletion of Tregs (p<0.001) in the tumor 3 days later (day 27) (Fig. 3D). As expected, peripheral Treg populations in the draining lymph nodes (Fig. 3E) and spleens (Fig. 3F) were also significantly reduced compared with isotype treated controls (p<0.001). Interestingly, we observed a 3 fold decrease in the absolute percentage of CD4^+^ T cells (Fig. 4A) and CD8a^+^ T cells (Fig. 4B) infiltrating into the tumor after administration of PC61. Furthermore, PC61 significantly depleted populations of CD4^+^ T cells (p<0.05, Fig. 4C) and CD8^+^ T cells (p<0.01, Fig. 4D) in the tumor draining lymph nodes. There was no change noted in the percentages of CD4^+^ T cells and CD8a^+^ T cells in the spleen (p>0.05, Fig. 4E–F) and MΦ and DC remained unchanged in tumors and draining lymph nodes although increased slightly in spleen (Fig. 5).

### Treatment with PC61 prevents activation of adaptive anti-tumor immune responses and inhibits tumor regression when used in combination with Ad-Flt3L and Ad-TK

**Figure 6:**
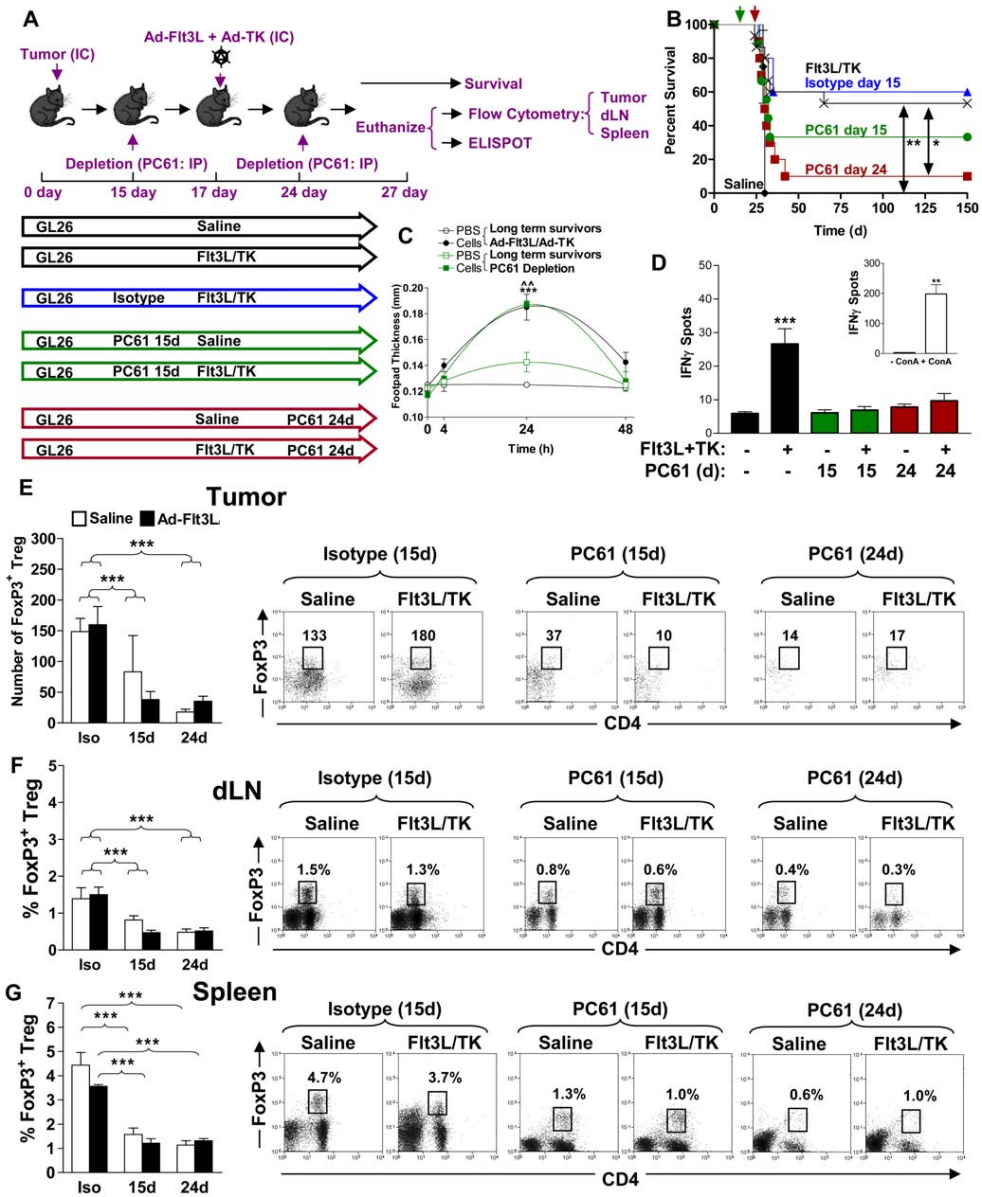
PC61 prevents activation of adaptive anti-tumor immune responses using Ad-Flt3L and Ad-TK. (A) C57BL/6 mice were challenged with tumor cells on day 0, and treated on day 17 by intratumoral delivery of Ad-Flt3L and Ad-TK or saline as indicated. Tregs were depleted on either day 15 (green) or day 24 (red) by ip injection of PC61 or on day 15 with the isotype control (blue). Immune cells were isolated from the spleen, the cervical LN and the tumor. Mice were either used for survival studies or euthanized on day 27 for analysis of immune cell populations by flow cytometry and ELISPOT. (B) Kaplan-Meier curve displaying survival data from 10 mice per group. Statistically significant differences to Ad-Flt3L and Ad-TK treated mice only (black line, no PC61 depletion) were calculated using the log-rank test (*p<0.05, **p<0.01). Administration of PC61 to mice 24 days after tumor implantation (7 days after treatment with Ad-Flt3L and Ad-TK) significantly reduced long term survival. (C) Tumor bearing mice were treated with Ad-Flt3L and Ad-TK alone (black lines) or with Ad-Flt3L and Ad-TK with PC61 depletion on day 15 (green lines). Long term survivors (60 d after tumor implantation) were assessed for a DTH response to irradiated GL26 cells injected into the right footpad (compared to PBS only in the left footpad). Two Way ANOVA with Tukey's post test was used to calculate significant differences between groups (***p<0.001 mice treated with Ad-Flt3L and Ad-TK no depletion, 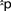
p<0.01 Ad-Flt3L and Ad-TK treated mice depleted with PC61 on day 15). (D) IFNγ ELISPOT showing the total number of IFNγ producing T lymphocytes that were stimulated with DC loaded with GL26 tumor extracts. T lymphocytes were purified from mice 10 d after treatment with either Ad-Flt3L and Ad-TK (+) or saline (−) as indicated. Black bars are T lymphocytes from mice that did not receive CD25 depleting immunoglobulins. Green bars are T lymphocytes purified from mice depleted with PC61 2 days before treatment with Ad-Flt3L and Ad-TK (day 15 after tumor implantation) and red bars are T lymphocytes purified from mice depleted with PC61 7 days after treatment with Ad-Flt3l and Ad-TK (day 24 after tumor implantation). (E–G) Tregs (CD3ε^+^ CD4^+^ Foxp3^+^) infiltrating tumors (E), draining lymph nodes (F) or spleens (G) were quantified in mice administered rat IgG1 isotype control (Iso) or PC61 on day 15 (15) or day 24 (24) as indicated in the graphs (left). Administration of PC61 to mice resulted in long term depletion of Tregs in peripheral sites (spleen and lymph nodes) and at the tumor. Dot plots (right) display representative means±SEM of Foxp3^+^ Tregs quantified from 5 mice per group. The total number of T regs in the tumors (E) and the percentages of T regs with respect to the total number of CD45+ cells in the draining lymph nodes (dLN) or spleen (F and G) are indicated in representative dot plots. Two Way ANOVA with Tukey's post test were used to determine significant differences (***p<0.001).

We also aimed to investigate the effects of depletion of Tregs using an anti-CD25 immunoglobulin on the efficacy of the T cell-dependent immunotherapy mediated by intratumoral delivery of Ad-Flt3L and Ad-TK [27], [37]. Activated CD4^+^ and CD8^+^ T lymphocytes upregulate cell surface CD25 after initial binding of the TCR with antigen displayed on MHC [30], [31] and this might explain the apparent decrease in T cells that was observed in tumors (Fig. 4A–B) and dLN (Fig. 4C–D) following administration of PC61. To investigate this further, and determine whether elimination of Tregs using PC61 could adversely affect T cell dependent immunotherapy of GBM, we implanted tumors in the striatum on day 0 and mice were treated with Ad-Flt3L and Ad-TK 17 days later, whilst PC61 or isotype control immunoglobulins were administered to groups on day 15 or day 24 (Fig. 6A). When we injected Ad-Flt3L and Ad-TK into the tumor on day 17 (when the tumor was 1mm^3^ in size (Fig. 1A) we observed a robust increase in survival with 50% of mice alive 150 d later (p<0.01, Fig. 6B) that was not adversely affected by administration of rat IgG1 isotype control (p>0.05). PC61 administered 2 days before injection of Ad-Flt3L and Ad-TK reduced survival to 30%, although this was not statistically different from isotype treated controls (p>0.05, Fig. 6B). However, when we treated mice with Ad-Flt3L and Ad-TK on day 17 and depleted Tregs on day 24, we found that long term survival was significantly reduced compared with mice that did not receive PC61 (p<0.05, Fig. 6B). Only 10% of tumor bearing mice survived 150 days later, suggesting that administration of PC6 17 d after intratumoral delivery of Ad-Flt3L and Ad-TK adversely affects T cell dependent brain tumor regression. In long term survivors after treatment with Ad-Flt3L and Ad-TK, a DTH response to irradiated GL26 cells in the footpad was observed when mice had been administered either PC61 (p<0.01) or isotype controls (p<0.001) (Fig. 6C). Moreover, treatment of tumor bearing mice with Ad-Flt3L and Ad-TK induced a robust increase in the total number of tumor responsive T cells that secreted IFNγ when co-incubated with BMDC loaded with GL26 cell extracts (p<0.001, Fig. 6D). Interestingly we did not observe any increase in IFNγ secreting T lymphocytes in response to BMDC loaded with GL26 cell extracts when PC61 was injected either prior to (15 days) or post (24 days) treatment using Ad-Flt3L and Ad-TK (p>0.05, Fig. 6D). The number of tumor infiltrating Tregs was reduced after systemic delivery of PC61 (p<0.001, Fig. 6E) and the percentage of Tregs was also significantly reduced in dLN and spleen, (p<0.001, Fig. 6F–G) irrespective of whether the mice were treated with saline or with Ad-Flt3L and Ad-TK.

### Treatment with PC61 prevents increases in tumor infiltrating T cells and MΦ but not DCs following treatment with Ad-Flt3L and Ad-TK

**Figure 7:**
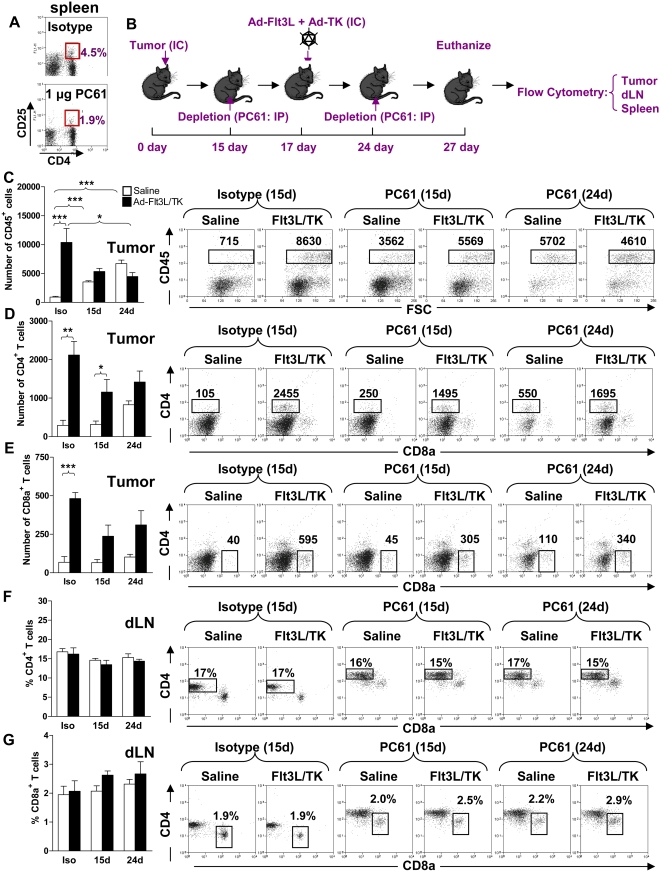
PC61 inhibits T cell clonal expansion in the tumors in response to treatment with Ad-Flt3L and Ad-TK. (A) Administration of PC61 to mice resulted in depletion of CD25^+^ T lymphocytes in the spleen compared with administration of isotype controls. (B) C57BL/6 mice were challenged with tumor cells on day 0, and treated on day 17 by intratumoral delivery of Ad-Flt3L and Ad-TK. Tregs were depleted on either day 15 or day 24 by ip injection of PC61. Immune cells were isolated from the spleen, the cervical LN, and the tumor at day 27 post-tumor implantation to determine the percentage of CD4^+^ and CD8a^+^ T cells in each tissue. (C–E) Total numbers of tumor infiltrating immune cells (CD45^+^) (C), CD4^+^ T cells (CD3ε^+^ CD4^+^ CD8a^−^) (D) and CD8a^+^ T cells (CD3ε^+^ CD4^−^ CD8a^+^) (E) and the percentage of CD4^+^ T cells (F) and CD8a^+^ T cells (G) in the draining lymph nodes of mice were determined. Panels on the right hand side (C–G) display representative dot plots for immune cells purified from tumor bearing mice treated with isotype rat IgG1 (day 15), PC61 (day 15) or PC61 (day 24) as indicated. The total number of CD45+ , CD4+ and CD8+ cells in the tumors are shown in representative dot plots (C–E). The percentages of CD4+ and CD8+ T cells with respect to the total number of CD45+ cells in the draining lymph nodes (dLN) are shown in representative dot plots (F and G). Two Way ANOVA with Tukey's post test was used to analyze differences between groups (C) and a two tailed t test was used to determine differences between saline and Ad-Flt3l/Ad-TK treated groups (D–G).

**Figure 8:**
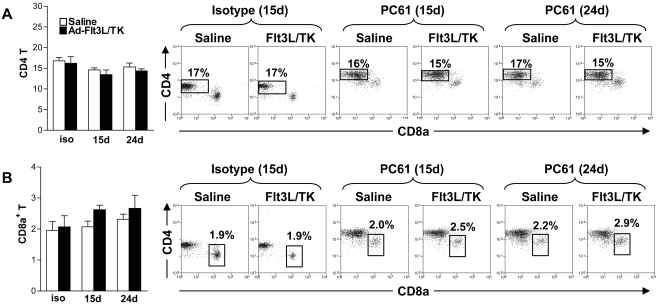
CD4+ and CD8a+ T cells do not change in the spleen either after PC61 depletion or in response to treatment with Ad-Flt3L and Ad-TK. Immune cells in the spleen were analyzed after tumor bearing C57BL/6 mice were treated with saline (white bars) or Ad-Flt3L and Ad-TK (black bars) and administered rat IgG1 isotype control immunoglobulins (-) on day 15 or PC61 on day 15 or day 24. Macrophages (A) CD4+ T cells (B) and CD8a+ T cells were assessed at day 27 post-tumor implantation using flow cytometry. Dot plots display CD8a against CD4 and are representative of between 5 and 10 mice per group. The percentages of CD4+ and CD8+ T cells with respect to the total number of CD45+ cells in the spleen are indicated in representative dot plots. Two Way ANOVA with Tukey's post test was used to determine statistical significance.

**Figure 9:**
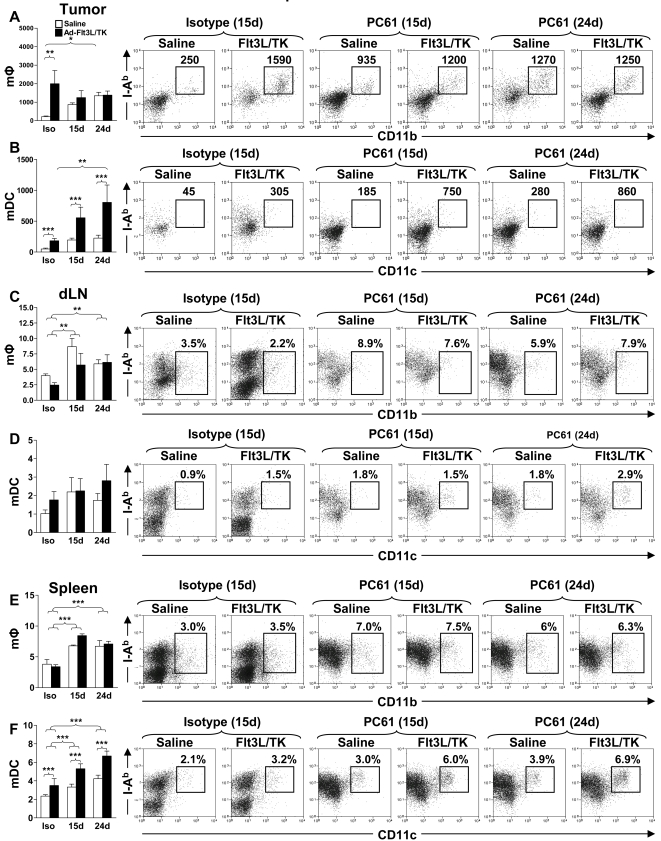
Antigen presenting cells increase in Tumor dLN and spleen after depletion with PC61 or in response to Ad-Flt3L and Ad-TK. GL26 cells were implanted in the striatum of C57BL/6 mice. Rat IgG1 isotype control immunoglobulins (-) were administered on day 15 and PC61 was administered on day 15 or day 24 as indicated. Mice were treated with intratumoral injection of Ad-Flt3L and Ad-TK (black bars) or saline (white bars). The percentage of tumor infiltrating macrophages (MΦ; CD11b+ CD45+ I-Ab+) (A,C,E) and mDC (mDC; CD11c+ CD45+ I-Ab+) (B,D,F) were quantified at day 27 post-tumor implantation using flow cytometry in the tumor (A,B) dLN (C,D) and spleen (E,F). 5 and 10 mice were analyzed per group. The total numbers of APCs in the tumors are indicated in representative dot plots (A–B). The percentage of APCs with respect to the total number of CD45+ cells in the draining lymph nodes (dLN) or spleen are depicted in representative dot plots (C–F). Two Way ANOVA with Tukey's post-test was used to determine statistical significance. (*p<0.05, **p<0.01, ***p<0.001).

In agreement with others, our data indicate that systemic delivery of PC61 causes the depletion of Foxp3^+^ Tregs [25], [33], [35]. Our results demonstrate that PC61 interferes with the induction of adaptive immune responses against brain tumor antigen. Further, administration of PC61 depleted 60% of spleen CD25^+^ T cells (Fig. 7A). Since clonal expansion of tumor antigen specific T cells induced by Ad-Flt3L-TK was inhibited by PC61 administration, we next determined whether PC61 affects the influx of CD4^+^ and CD8a^+^ T cells into the tumor, the draining lymph nodes and the spleen after treatment with Ad-Flt3L and Ad-TK (Fig. 7 B). The total number of CD45^+^ tumor infiltrating immune cells increased 10 d after intratumoral delivery of Ad-Flt3L and Ad-TK (p<0.001, Fig. 7 C), as did the numbers of CD4^+^ T cells (p<0.001, Fig. 7 D) and CD8a^+^ T cells (p<0.001, Fig. 7 E). This observation was consistent with the induction of adaptive immune responses against tumor antigen by intratumoral delivery of Ad-Flt3L and Ad-TK. Co-administration of PC61 either on day 15 or day 24, however, completely blocked the Ad-Flt3L and Ad-TK dependent increase in total numbers of tumor infiltrating immune cells (p>0.05, Fig. 7 C), and CD8a+ T cells (p>0.05, Fig. 7 E). Increased tumor infiltrating CD4+ T cells following treatment with Ad-Flt3L and Ad-TK was only inhibited when PC61 was administered on day 24 (p>0.05, Fig. 7 D). No changes were observed in the number of T cells in the dLN (Fig. 7 F–G) or spleen (Fig. 8). Moreover, increase in numbers of tumor infiltrating macrophages (p<0.01, Fig. 9 A) was also blocked by PC61 administration but infiltration of mDCs into tumors after administration of Ad-Flt3L and Ad-TK (p<0.001, Fig. 9B) was not suppressed. Together, our data suggest that infiltration of mDC was an early event, probably induced by expression of Flt3L, whereas infiltration of T cells occurred later and was dependent on the successful initiation of adaptive immune responses against tumor antigens. MΦ increased in dLN and spleen, while mDC only increased significantly in the spleen (Fig. 9 C–F).

## Discussion

Elimination of Tregs using depleting antibodies in combination with existing immunotherapies has been proposed to enhance anti-tumor immunity [38]. Herein we show in a syngeneic mouse model of GBM that depletion of Tregs using PC61, a CD25 specific immunoglobulin can also deplete activated T cells, and in doing so can interfere with the clonal expansion of tumor antigen specific T cells during immunotherapy. In agreement with previous reports [23], [24], [33], [35], [39], [40], we observed that tumors, and dLN were heavily infiltrated with CD25^+^ T cells. Previously, it was shown that administration of 250 µg CD25-depleting immunoglobulins 3 days before tumor implantation [32] and long-term administration of CD25-depleting immunoglobulins after tumor implantation (0.5 µg/injection, beginning 1 week after tumor implantation and given three times per week for 3 weeks) [25] can induce long term survival in about half of GL261 tumor bearing C57BL/6 mice. To determine the role of Tregs in tumor regression in a clinically relevant scenario of a large, pre-established tumor mass, we gave a single dose of PC61 hybridoma at two time points, either day 15 when the tumor was approximately 0.5mm^3^ or day 24 when the tumor was 15mm^3^. Herein we show that elimination of CD25^+^ T lymphocytes by a single injection of PC61 15 days after tumor implantation mediated tumor regression and improved long term survival in almost 50% of mice. However, Treg depletion increased the survival of tumor bearing mice only if depletion was done when the tumors were very small, i.e., 0.45mm^3^. Our findings are in agreement with other reports that show an anti-tumor effect of T reg depletion in mouse models of neuroblastoma [41], leukemia, fibrosarcoma, myeloma [35], and glioma [25], [32]. We observed that the antitumor effects of Treg depletion were not mediated by a T cell dependent anti-tumor immune response. Our results show that Treg depletion did not generate tumor-specific T cells as assed by ELISPOT assay nor did it induce anti-tumor immunological memory. These data suggest that the innate immune system could play a role in the anti-tumor effect elicited by Treg depletion in this model. In fact, T regs have been shown to inhibit the activity of the innate immune system by directly suppressing the cytotoxic effect of NK cells [42]. This inhibitory mechanism is dependent on TGF-β and cell-cell contacts [42]–[45]. Since we found that NK cells comprise ∼20% of the immune cells that infiltrate GL26 intracranial tumor, the cytotoxic activity of these cells could be involved in the antitumor effects observed after T reg depletion at day 15 after tumor implantation. Interestingly, no increase in survival was observed when PC61 was administered to tumor bearing mice 24 days after tumor implantation, this was probably due to the size of the tumor and the relatively short life expectancy of these mice (∼5 d to death). It is likely that the tumor burden will be an important factor in the clinic when patients are treated by depletion of Tregs, we would expect from these data that Treg depletion might show efficacy when treating minimal, residual tumors.

Further, we wished to determine whether depletion of Tregs with CD25-specific immunoglobulins would affect survival when implemented in combination with intratumoral delivery of Ad-Flt3L and Ad-TK. Along these lines, it has been reported that depletion of Tregs using PC61 improved survival when GBM bearing mice were vaccinated with DC transfected with tumor cell mRNA [26]. However, that study assessed the prophylactic, not therapeutic effects of PC61 against brain tumors; since tumor cells were implanted 10 days after depletion of Tregs and 7 days after vaccination with DC pulsed with tumor cell mRNA [26]. In our model, tumors were already well established (17 days) prior to intratumoral delivery of Ad-Flt3L and Ad-TK to induce T cell-dependent tumor regression. Administration of PC61, 2 days before treatment (15 days after tumor implantation) induced tumor regression in about 50% of mice which was not significantly different to treatment with Ad-Flt3L and Ad-TK alone. However, when PC61 was administered 7 days after injection of Ad-Flt3L and Ad-TK, therapeutic improvement in survival was almost completely abolished, due to the depletion and/or functionally inactivated tumor antigen specific T lymphocytes that had become activated in response to the therapy. Our results highlight that depletion of Tregs by targeting CD25 might interfere with the induction of adaptive immune responses against brain tumor antigen.

A cell surface marker specific for Tregs that could be targeted with immunoglobulins to specifically deplete Tregs would beneficial to overcome immunological tolerance/ignorance to brain tumor antigens. Unfortunately, the cell surface proteins on Tregs (for example; CD25, CTLA4) are also expressed on other immune cells such as activated T lymphocytes [46]. CTLA4 has also been widely studied as a target to specifically deplete Tregs. Intracellular CTLA4 is expressed at high levels in Tregs and is also expressed at much lower levels on the cell surface of resting Tregs. Activation of Tregs stimulates the upregulation of CTLA4 on their cell surface and may thus be a useful target to specifically inhibit Tregs. Depletion of Tregs using immunoglobulins against CTLA4 has been found to improve survival in mouse models of glioma [47]. CTLA4 is also expressed in the ER of helper and effector T lymphocytes and cell surface expression is upregulated 24 to 48 h after activation of TCR [48]. It remains to be investigated, however, whether CTLA4 depletion might also interfere with the clonal expansion of tumor-antigen specific T lymphocytes.

Our results suggest that while elimination of Tregs might be sufficient to eliminate smaller tumors, the depletion of Tregs using CD25-targetting strategy might interfere with the clonal expansion of tumor antigen specific T lymphocytes if used in combination with another immunotherapeutic strategy. Foxp3 is currently the only marker that has been identified that is exclusively expressed on Tregs and not on other immune cells. However, Foxp3 is intranuclear and therefore cannot be easily depleted using immunoglobulins. Further study on the mechanism of action of Foxp3 and/or cell surface Treg specific markers might highlight molecular targets that could be useful to specifically eliminate Tregs. Our results indicate that clinical trials which propose to utilize CD25 depleting antibodies, or similar Treg depleting approaches, must carefully monitor the effects on normal activated T cells, and adjust dosing schedules accordingly. Further, the use of Treg depleting strategies will only be useful in the context of minimal residual disease.

## Materials and Methods

### Adenoviral vectors, Cell Lines, plasmids, and reagents

We used first generation, recombinant adenoviral vectors (serotype 5) expressing Herpes Simplex Virus Type I Thymidine Kinase (Ad-TK) [27], [29] and Fms-like tyrosine kinase 3 ligand (Ad-Flt3L)[28], [49] in this study. The methods for adenoviral generation, purification, characterization and scale up have been previously described by our lab[27], [29], [49]. GL26 cells were obtained from ATCC (Manassas, VA) and were grown in DMEM culture media supplemented with 10% FCS and 1% Pen-Strep and passaged routinely every 2-3 days. Ascites fluid from the PC61 hybridoma was purchased from BioExpress Cell Culture Services (West Lebanon, NH) and isotype rat IgG1 control was purchased from Sigma (St Louis MO). Foxp3-FITC was purchased from ebioscience, primary antibodies for immunofluorescence and immunohistochemistry (CD45, F4/80, CD205, CD4, CD8, CD19, CD25 and GFAP) were all purchased from Serotec and biotinylated secondary antibodies were purchased from Dako (Denmark) and fluorescent tagged secondary antibodies were purchased from Invitrogen (Carlsbad, CA). All antibodies used for flow cytometry were purchased from BD Pharmingen (San Diego, CA). Recombinant human Annexin V-FITC was purchased from BenderMedSystems (Vienna, Austria) and the BrdU DNA labeling and detection kit was procured from Exalpha Biologicals Inc (Maynard MA). All other chemicals were purchased from Sigma.

### Mouse glioblastoma model

Wild type C57BL/6J mice were purchased from Jackson Laboratories (Bar Harbor, ME, USA). To establish a syngeneic tumor model, we used GL26 cells that were derived from a chemically induced glioma in a female C57BL/6 mouse. Female mice (6–12 weeks) were anesthetized with an IP injection of Ketamine (75 mg/kg) and Medetomidine (0.5 mg/kg), placed in a stereotactic apparatus modified for mice, and a hole was drilled in the skull. 20,000 GL26 cells in 0.5 µl PBS were deposited unilaterally into the right striatum (+0.5 mm AP, +2.2 mm ML, −3.0 mm DV from bregma) using a 5 µl Hamilton syringe with a 33-gauge needle. The needle was left in place for 3 minutes prior to removal to allow tumor cells to settle at the injection site. Mice were resuscitated using atipamazole (IP) and administered buprinex (SQ dose) as an analgesic. 17 days after tumor implantation and using the same stereotactic coordinates, mice received an intratumoral injection of 5×10^8^ iu Ad-Flt3L and 5×10^7^ iu Ad-TK in 1 µl saline. Alternatively, mice were injected with 1 µl saline as a control. Mice treated with Ad-Flt3L and Ad-TK also received 25 µg/kg GCV (IP, Roche, Boulder CO) twice daily for 7 days starting the day after vector injection. CD25^+^ Tregs were depleted by a single ip injection of 1mg ascites fluid (600 µl) from the PC61 hybridoma. Alternatively GL26 tumors were implanted into female T reg deficient athymic nude mice (Simonsen labs) and PC61 or control IgG was administered ip 15 days later as described. All animals were housed in specific pathogen free environment, and were closely monitored All animal experiments were performed after prior approval by the Institutional Animal Care and Use Committee at Cedars-Sinai Medical Center and conformed to the policies and procedures of the Cedars-Sinai Medical Center Comparative Medicine Department. Mice used in this study were monitored for signs of moribund behavior and euthanized when their health status reached criteria established by the guidelines of the IACUC. Animals were euthanized at first signs of moribund behavior, by terminal perfusion with oxygenated, heparinized Tyrode's solution (132 mM NaCl, 1.8 mM CaCl_2_, 0.32 mM NaH_2_PO_4_, 5.56 mM glucose, 11.6 mM NaHCO_3_, and 2.68 mM KCl) and were fixed with 4%PFA in PBS for immunofluorescence and immunohistochemistry.

### Immunohistochemistry and Immunofluorescence

Fifty micrometer-thick coronal sections were cut through the striatum using a vibratome. Free-floating immunohistochemistry was performed to detect inflammatory and immune cell markers. Endogenous peroxidase was inactivated with 0.3% hydrogen peroxide (only for IHC). Sections were subjected to antigen retrieval by incubation in 10mM Citrate at 60°C for 10 minutes before washing and blocked with 10% horse serum (Life Technologies, Carlsbad, CA) in TBS+0.5% Triton X-100 (Sigma) before incubating for 72 h with primary antibodies CD45 (1∶1000), F4/80 (1∶1,000), CD205 (1∶500), CD4 (1∶1000), CD8a (1∶5,000), CD19 (1∶1000), CD25 (1∶500) and GFAP (1∶1000). Biotinylated secondary antibodies for IHC (Dako) were incubated with sections (1∶1,000 in 1% horse serum in TBST) for 4 h and detected using the Vectastain Elite ABC horseradish peroxidase method (Vector Laboratories, Burlingame, CA). Immunoreactive cells were developed with diaminobenzidone (Sigma) and glucose oxidase (Sigma). Sections were mounted on gelatinized glass slides and dehydrated through graded ethanol solutions. Secondary antibodies for IF (Alexa-488 conjugated) were purchased from Molecular probes and sections were stained for 4 h (1∶1000 in 1% horse serum in TBST). Sections were stained with DAPI (1 µg/ml in PBS) for 20 minutes and mounted on glass slides. Prolong Gold anti-fade reagent (Invitrogen) was used to attach coverslips. A Zeiss Axioplan 2 microscope was used to visualize IHC staining.

### Flow Cytometry

To isolate tumor infiltrating immune cells, mice were perfused with 100 ml heparinized Tyrode's Solution 7 days after intratumoral injection of virus or saline. Brains were separated from the meninges and removed from the skull. The tumor was carefully dissected and removed, taking care to avoid the ventricles. The tumor tissue was then diced with a razor blade before homogenizing in RPMI medium using a glass Tenbroeck homogenizer. Mononuclear cells were purified from brain tissue by centrifugation (600xg) through a Percol gradient; mononuclear cells migrate to the interface between 30% and 70% Percol. Cells were counted and labeled with antibodies for analysis by flow cytometry. Splenocytes were harvested and removed of red blood cells using ACK solution (0.15 mM NH_4_Cl, 10 mM KHCO_3_, and 0.1 mM disodium EDTA at pH 7.2). Lymph nodes were homogenized in a Tenbroeck homogenizer. Immune cells were labeled with antibodies in cell surface staining buffer (0.1M PBS, w/o Ca^++^, Mg^++^, with 1% FBS, 0.1% Sodium Azide) for analysis by flow cytometry using a FACScan flow cytometer (Beckton Dickenson). To analyze Foxp3+ Tregs (CD3^+^ CD4^+^ Foxp3^+^), we stained with CD3-PE, CD4-PerCP and Foxp3-FITC. CD25^+^ T cells were stained with CD3-PE, CD4-PerCP, CD25-Biotin and streptavidin-FITC (BD-Pharmingen). mDC (CD11c^+^ MHC II^+^ CD45^+^) were stained with CD11c-PE, MHC II-FITC and CD45-PerCP. Macrophages (CD11b^+^ MHC II^+^ CD45^+^) were stained with CD11c-PE, MHC II-FITC and CD45-PerCP. CD4^+^ and CD8a^+^ T lymphocytes were stained with CD3-PE, CD4-PerCP and CD8a-FITC. Other immune cells analyzed during this study (not shown) were NK cells (CD3^−^ CD161^+^ CD45^+^), NK-T cells (CD3^+^ CD161^+^ CD45^+^), and B lymphocytes (CD3^−^ CD19^+^ CD45^+^). Analysis was performed using between 5 and 10 mice per group. All percentages were calculated based on the total number of CD45^+^ immune cells in the tumor. For example, to calculate the percentage of mDC in the tumor, the total number of gated mDC (CD11c^+^ MHCII^+^ CD45^+^) were counted and the percentage of mDC in the tumor was determined by dividing the total number of mDC by the total number of CD45^+^ cells in the tumor. In Fig. 1 E and F we calculated the percentage of Tregs based on the total number of T cells (number of CD25+CD4+ cells/number of CD3ε+ cells).

### Annexin V vs Propidium Iodide staining

Annexin V-FITC and propidium iodide were used to detect apoptosis by flow cytometry as described previously [50]. Briefly cells were growin in 96 well plates (20,000 cells per well) and incubated with 100 µg/ml PC61 or 100 µg/ml rat IgG1 isotype control for 48 h. Cells were then harvested gently using Trypsin and resuspended in 100 µl Annexin V Binding Buffer (150mM NaCl, 18mM CaCl_2_, 10mM HEPES, 5mM KCl, 1mM MgCl_2_). Cells were incubated at room temperature (21 °C) for 5 minutes in the dark, before adding propidium iodide (5 µg/ml) for 1 minute. Cells were analyzed on a FACScan flow cytometer (BD Biosciences). Cell Quest software (BD Biosciences) was used to analyze the data.

### BrdU labeling and detection

GL26 cell proliferation was assessed using a BrdU colorimetric ELISA (ExAlpha Biologicals, Inc.). Briefly, GL26 cells were incubated in 96 well plates at 20,000 cells per well. 100 µg/ml PC61 or the rat IgG1 isotype control were added to wells and cells were incubated for 24 h. 1x BrdU was added to the wells for 4 h and BrdU incorporation into newly synthesized DNA strands was detected exactly as recommended by the manufacturer.

### ELISPOT

10^5^ splenocytes from tumor bearing mice (wild type C57BL/6 or *TLR2^−/−^*) treated with saline or Ad-Flt3L and Ad-TK were prestimulated on a monolayer of GL26 cells (fixed in 1% PFA for 15 min at RT) in the presence of 10 U/ml IL-2 for 14 days. Fresh IL-2 was added every 4^th^ day. BMDC (CD11c+, CD45+, MHC II +) were cultured as described previously[51]. Briefly, bone marrow was flushed out from the long bones of mice using 0.5 ml RPMI media. A single cell suspension was created by pipetting up and down with a P1000 pipette. BMDC were cultured from hematopoietic stem cell precursors by supplementing the media with 10 ng/ml Flt3L conditioned media. Fresh GMCSF and IL4 (10ng/ml each) was added every 2–3 days. By day 7, >90% of loosely adherent cells were CD11c^+^ (data not shown). Loosely adherent cells were removed and were loaded with 100 µg/ml GL26 cell extract (prepared fresh by freeze/thawing) for 6 h at 37 °C before washing 3 times with PBS. 1×10^5^ stimulator cells (BMDC loaded with GL26 or LLc1 cell extract) were incubated with 1×10^5^ responder cells (prestimulated T cells) in triplicate for 48 h on precoated 96 well mouse IFNγ ELISPOT plates. IFNγ spots were developed as recommended by the manufacturer's detailed instructions (R&D Systems). ConA (1ug/ml, 4 h) stimulation of WT and TLR2-/- splenocytes was used as a control.

### Delayed Type Hypersensitivity (DTH) Test

DTH test was performed on long-term survivor mice 60 d after tumor implantation as follows. 50,000 irradiated GL26 cells (30 Gy using a ^137^Cs source) were injected in 100 µl PBS in the right rear footpad (right) or 100 µl PBS control in the left rear footpad (left) of mice. The thickness of the footpad (left and right) was recorded 0 h (immediately prior to depositing cells in the footpad), 4 h, 24 h and 48 h after injection of irradiated cells or PBS using a vernier microcalipers.

### Statistical Analysis

Kaplain-Meier survival curves were analyzed using the logrank test (GraphPad Prism Version 3.03). One- or Two-way ANOVA followed by Tukey's test or unpaired two-tailed Student's *t* test were used to analyze all other data as indicated in the results and the figure legends (NCSS software; NCSS, Kaysville, UT). When data failed normality test or Levene equal-variance test, they were square-root transformed. Results were expressed as the mean±SEM. A p value <0.05 was considered the cut off for significance. All experiments were performed at least twice.
